# PI 3-kinase and disease

**DOI:** 10.1186/1753-6561-6-S3-O2

**Published:** 2012-06-01

**Authors:** Lewis C Cantley

**Affiliations:** 1 Beth Israel Deaconess Medical Center and Harvard Medical School, Boston MA 02115, USA

## 

Phosphoinositide 3-Kinase (PI3K) is a central enzyme in a signaling pathway that mediates cellular responses to growth factors. This enzyme phosphorylates the 3 position of phosphatidylinositol-4,5-bisphosphate to produce phosphatidylinositol-3,4,5-trisphosphate (PIP_3_) at the plasma membrane. A number of signaling proteins, including the Ser/Thr protein kinases, AKT and PDK1, contain pleckstrin homology domains that bind specifically to PIP_3_. Thus, the generation of PIP_3_ at the plasma membrane in response to activation of PI3K by growth factors results in the initiation of downstream Ser/Thr phosphorylation cascades that control a variety of cellular responses. The signaling pathway downstream of PI3K is highly conserved from worms and flies to humans and genetic analysis of the pathway has revealed a conserved role in regulating glucose metabolism and cell growth. Based on deletion of genes encoding the catalytic or regulatory subunits of PI3K in the mouse, PI3K mediates insulin dependent regulation of glucose metabolism, and defects in activation of this pathway result in insulin resistance. In contrast, mutational events that lead to hyperactivation of the PI3K pathway result in cancers. Activating mutations in PIK3CA, encoding the p110alpha catalytic subunit of PI3K or inactivating mutations in PTEN, a phosphoinositide 3-phosphatases that reverses the effects of PI3K, are among the most common events in solid tumors. We have generated mouse models in which a mutated form of the PIK3CA gene is expressed in a tissue specific and reversibly inducible manner. These mice develop cancers that are dependent on continuous expression of the mutant PIK3CA gene. The PIK3CA driven tumors are FDG-PET positive and turning off PI 3-Kinase with PI3K inhibitors that are in human clinical trials results in an acute decline in FDG-PET signal that precedes tumor shrinkage. These results suggest that the ability of PI3K to stimulate high rates of glucose uptake and metabolism may be critical for the survival of PIK3CA mutant tumors. The role of PI3K inhibitors for treating cancers in mouse models and in human trials will be discussed.

